# Tree Shrew Cells Transduced with Human CD4 and CCR5 Support Early Steps of HIV-1 Replication, but Viral Infectivity Is Restricted by APOBEC3

**DOI:** 10.1128/JVI.00020-21

**Published:** 2021-07-26

**Authors:** Meng-Ting Luo, Dan Mu, Xiang Yang, Rong-Hua Luo, Hong-Yi Zheng, Min Chen, Ying-Qi Guo, Yong-Tang Zheng

**Affiliations:** a Key Laboratory of Animal Models and Human Disease Mechanisms of the Chinese Academy of Sciences/Key Laboratory of Bioactive Peptides of Yunnan Province, KIZ-CUHK Joint Laboratory of Bioresources and Molecular Research in Common Diseases, Center for Bio-safety Mega-Science, Kunming Institute of Zoology, Chinese Academy of Sciences, Kunming, Yunnan, China; b Kunming College of Life Science, University of Chinese Academy of Sciences, Kunming, Yunnan, China; c Institute of Life Sciences, Chongqing Medical University, Chongqing, China; d National Resource Center for Non-Human Primates, and National Research Facility for Phenotypic & Genetic Analysis of Model Animals (Primate Facility), Kunming Institute of Zoology, Chinese Academy of Sciences, Kunming, Yunnan, China; Icahn School of Medicine at Mount Sinai

**Keywords:** APOBEC3, HIV-1, restriction factors, tree shrews, *Tupaia belangeri chinensis*, animal model

## Abstract

The host range of human immunodeficiency virus type 1 (HIV-1) is narrow. Therefore, using ordinary animal models to study HIV-1 replication, pathogenesis, and therapy is impractical. The lack of applicable animal models for HIV-1 research spurred our investigation on whether tree shrews (Tupaia belangeri chinensis), which are susceptible to many types of human viruses, can act as an animal model for HIV-1. Here, we report that tree shrew primary cells are refractory to wild-type HIV-1 but support the early replication steps of HIV-1 pseudotyped with the vesicular stomatitis virus glycoprotein envelope (VSV-G), which can bypass entry receptors. The exogenous expression of human CD4 renders the tree shrew cell line infectible to X4-tropic HIV-1_IIIB_, suggesting that tree shrew CXCR4 is a functional HIV-1 coreceptor. However, tree shrew cells did not produce infectious HIV-1 progeny virions, even with the human CD4 receptor. Subsequently, we identified tree shrew (ts) apolipoprotein B editing catalytic polypeptide 3 (tsAPOBEC3) proteins as active inhibitors of HIV-1 particle infectivity, with virus infectivity reduced 10- to 1,000-fold. Unlike human APOBEC3G, the tsA3Z2c-Z1b protein was not degraded by the HIV-1 viral infectivity factor (Vif) but markedly restricted HIV-1 replication through mutagenicity and reverse transcription inhibition. The pooled knockout of tsA3Z2c-Z1b partially restored the infectivity of the HIV-1 progeny. This work suggests that tsAPOBEC3 proteins serve as an additional barrier to the development of HIV-1 tree shrew models, even when virus entry is overcome by exogenous expression of human CD4.

**IMPORTANCE** The development of animal models is critical for studying human diseases and their pathogenesis and for evaluating drug and vaccine efficacy. For improved AIDS research, the ideal animal model of HIV-1 infection should be a small laboratory mammal that closely mimics virus replication in humans. Tree shrews exhibit considerable potential as animal models for the study of human diseases and therapeutic responses. Here, we report that human CD4-expressing tree shrew cells support the early steps of HIV-1 replication and that tree shrew CXCR4 is a functional coreceptor of HIV-1. However, tree shrew cells harbor additional restrictions that lead to the production of HIV-1 virions with low infectivity. Thus, the tsAPOBEC3 proteins are partial barriers to developing tree shrews as an HIV-1 model. Our results provide insight into the genetic basis of HIV inhibition in tree shrews and build a foundation for the establishment of gene-edited tree shrew HIV-1-infected models.

## INTRODUCTION

The development of animal models is essential for studying the pathogenesis of AIDS and for evaluating the efficacy of drugs and vaccines. As the main pathogen of AIDS, human immunodeficiency virus type 1 (HIV-1) has a limited host range, with spreading replication found exclusively in humans (Homo sapiens) and chimpanzees (Pan troglodytes) (1). The use of chimpanzees in research is difficult due to ethical and financial considerations, and HIV-1 shows low morbidity in infected chimpanzees. Although northern pig-tailed macaques (Macaca leonina) can be infected with HIV-1, including HIV-1 with viral infectivity factor (Vif) from simian immunodeficiency virus (SIV), they often exhibit long-term low-level replication, latent infection, and low morbidity *in vivo* (2–5). Thus, there is no perfect primate model or small animal model currently available for HIV-1 investigations (6, 7). Transgenic mouse systems containing non-HIV promoters have been developed to model HIV-1 infection during the postintegration phase (8). However, despite exhibiting efficient HIV-1 infection, severely immunodeficient mice lack humoral and cellular responses to the virus (9, 10), thereby limiting their application in HIV/immune system research. Therefore, the potential usefulness of an animal with an intact immune system as a model of HIV-1 infection warrants further assessment of the limitations and impediments of the viral life cycle in these hosts (9, 10).

Tree shrews (Tupaia belangeri chinensis) exhibit considerable potential as an animal model for studying human diseases and therapeutic responses (11–13). Due to their unique characteristics, including small body size, short reproductive cycle, short life span, low-cost maintenance, and close affinity to primates (11, 14), tree shrews have been employed as animal models of viral infection in various research (14–16). Although tree shrews are not commonly studied due to a lack of pure inbred animals, limited access to animal resources, and inadequate references, this species has attracted increasing attention as a viable alternative to primates in biomedical research and drug safety testing (17). Of note, tree shrews have shown susceptibility to infection with hepatitis B (18–21), hepatitis C (14, 15), influenza (22), herpes simplex (23), coxsackie A16 (24), dengue (25), and Zika viruses (16).

Successful HIV-1 replication requires the completion of multiple steps. First, cell surface chemokine receptors, namely, the CD4 receptor and CCR5 or CXCR4 coreceptors, mediate membrane fusion and viral entry. Subsequently, the virus undergoes proper uncoating, followed by reverse transcription of viral RNA into double-stranded DNA, which is then imported into the nucleus and integrated into the host genome. After that, the viral genome produces proteins and RNA that are packaged together for a further round of infection. During these steps, host cells can block the early and late stages of HIV-1 replication by the expression of restriction factors, which is one of the main species-specific barriers for the development of HIV-1/AIDS animal models.

Host restriction factors such as tripartite motif protein 5α (TRIM5α), apolipoprotein B-editing catalytic polypeptide 3G (APOBEC3), SAM domain- and HD domain-containing protein 1 (SAMHD1), and tetherin and myxovirus resistance 2 (Mx2) limit distinct replication stages of retroviruses (26, 27). Previous research on TRIM5 proteins revealed a novel mode of non-self-recognition that protects against cross-species transmission of retroviruses (28). In simian cells, the TRIM5α protein induces the premature capsid uncoating of HIV-1 but not of SIV (29). The APOBEC3 family of proteins restricts HIV-1 by the deamination of cytosine residues to uracil in single-stranded DNA (ssDNA) during reverse transcription in HIV-1 particles (26) or by other deamination-independent functions (30–32). Based on high-quality genome sequences and annotation (11, 33, 34), a series of immune genes have been identified in tree shrews recently (35–40). Earlier research showed that retinoic acid-inducible gene I (*RIG-I*), which encodes an intracellular pattern recognition receptor (PRR) in the innate immune system that participates in viral RNA recognition, is lost in the Chinese tree shrew lineage (40). Although melanoma differentiation factor 5 (*MDA5*), which is a PRR and shares similar signaling features and structural homology as *RIG-I*, can functionally substitute for *RIG-I* in tree shrews to sense RNA viruses and induce type-1 interferon (*IFN-1*) response (37, 40), delayed *IFN-1* expression renders tree shrews more susceptible to viral infection. Our previous work showed that tree shrew TRIM5 proteins, including TRIMCyp, are unable to restrict HIV-1, unlike rhesus monkey TRIM5, which impacts macaque HIV-1 models by delivering potent anti-HIV-1 activity (39). In addition, we identified five tree shrew (ts) APOBEC3 family proteins that induce G-to-A hypermutation in HIV-1 genome DNA (35). However, whether HIV-1 Vif can degrade tsAPOBEC3 proteins, as it does to human APOBEC3, remains unclear. Investigating this could help determine if tree shrews can serve as a potential HIV-1 animal model.

Here, we showed that tree shrew primary cells are refractory to replication-competent HIV-1 but support vesicular stomatitis virus glycoprotein envelope (VSV-G)-HIV-1-green fluorescent protein (GFP) infection, which bypasses the cell membrane receptors. The exogenous expression of human CD4 or CD4/CCR5 in tree shrew lung fibroblasts (TSLFs) also supports HIV-1 entry and efficient infection. However, the virions produced from the human-receptor-expressing TSLFs exhibit low infectivity. Furthermore, the tsAPOBEC3 proteins are potent inhibitors of HIV-1 infection via the insertion of hypermutations and restriction of reverse transcription. Moreover, tsA3Z2c-Z1b is not degraded by HIV-1 Vif. Hence, multiple restrictions exist against HIV-1 replication in tree shrew cells. Our study provides information regarding intrinsic immune restriction factors in tree shrews and essential guidance for developing a tree shrew HIV-1 infection model.

## RESULTS

### Primary tree shrew cells can be infected by HIV-1 (VSV-G).

**FIG 1:**
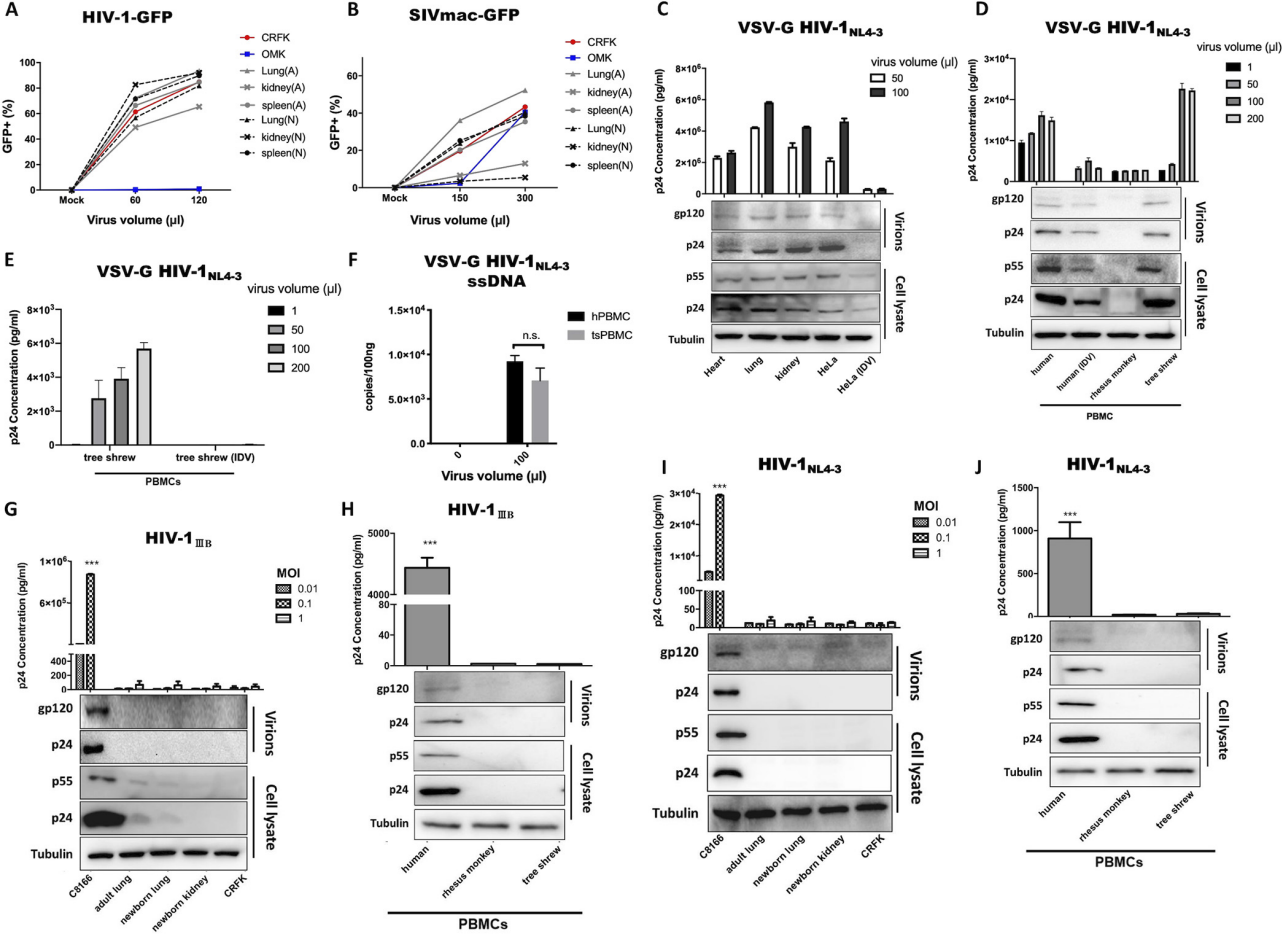
Infection of primary tree shrew cells with VSV-G HIV-1 but not with wild-type HIV-1. (A and B) Primary tree shrew cells (e.g., lung, spleen, heart, and kidney from adult [marked as A] or newborn [marked as N] tree shrews) were subjected to infection with various doses of single-cycle VSV-G-pseudotyped HIV-1-GFP virus (A) and SIVmac-GFP virus (B). (C and D) At 3 dpi, percentages of GFP-positive cells were determined by flow cytometry. Tree shrew primary cells (C) and tree shrew, human, and rhesus monkey PBMCs (D) were infected with VSV-G HIV-1_NL4-3_. Expression levels of p24 in supernatants were then analyzed at 3 dpi by ELISA. (E) tsPBMCs were infected by VSV-G HIV-1_NL4-3_ with IDV. At 30 hpi, expression of p24 in supernatants was analyzed. (F) At 4 hpi with VSV-G HIV-1_NL4-3_, ssDNA signals were detected by RT-qPCR in PBMCs. (G to J) HIV-1_IIIB_ was used to infect primary tissue cells (G) or PBMCs (H); HIV-1_NL4-3_ was used to infect primary tissue cells (I) or PBMCs (J), with p24 expression levels in the supernatant analyzed at 3 dpi. Virus proteins of infected cells and virions (infected with highest virus volume or MOI) were detected by Western blotting. HeLa (IDV) and human (IDV) are HIV-1 protease inhibitor indinavir controls, which were added to confirm the infection system.

To evaluate tree shrew potential as an animal model of HIV-1, we first determined if tree shrew cells could support the early phases of HIV-1 replication. Primary tree shrew cells (e.g., lung, spleen, heart, and kidney cells from adult or newborn tree shrews) were subjected to infection with single-cycle VSV-G-pseudotyped HIV-1-GFP virus, which bypasses the HIV-1 cellular receptor. The Crandell-Rees feline kidney (CRFK) cell line was used as a positive control, and owl monkey (Aotus trivirgatus) kidney (OMK) epithelial cells, which express nonpermissive TRIMCyp, were used as a negative control (41). At 3 days postinfection (dpi), the percentage of GFP-positive cells was examined to indicate the permissivity of infection. As shown in Fig. 1A, all primary cells from adult and newborn tree shrews had GFP percentages similar to that in CRFK cells, whereas almost no OMK cells were GFP positive. In addition, the primary tree shrew cells were permissive for SIV-GFP expression (Fig. 1B). The GFP signal percentage was high in splenic and lung cells but low in kidney cells.

To further confirm whether primary tree shrew cells were susceptible to retrovirus transduction, VSV-G HIV-1_NL4-3_, a full-length replication-competent HIV-1, was used to infect tree shrew cells. Again, primary cells from the heart, lung, and kidney were used, and HeLa cells with or without HIV-1 protease inhibitor indinavir (IDV) 100 nM were used as controls to confirm the infection system. Results showed that HIV-1 p24 proteins were as efficiently expressed in the culture of primary tree shrew cells as that of HeLa cells (Fig. 1C). Furthermore, tree shrew peripheral blood mononuclear cells (PBMCs) produced HIV-1 p24 equivalent to that found in human PBMCs (Fig. 1D), suggesting that the long terminal repeat (LTR)-driven transcription of HIV-1 was processed efficiently in the tree shrew cells. The expression levels of virus protein p24 in cells and virions produced from cells infected with the highest virus volume were detected by Western blotting. Results showed that IDV limited HIV-1 expression in the tree shrew (ts) PBMCs (Fig. 1E). Whole DNA was then extracted from these cells for the detection of reverse transcription (ssDNA) using real-time quantitative PCR (RT-qPCR). As shown in Fig. 1F, the tsPBMCs displayed comparable ssDNA signals as those of the human PBMCs. This ensured that the VSV-G HIV-1_NL4-3_ virus entered the tree shrew cells and successfully commenced reverse transcription.

Whether primary tree shrew cells or PBMCs can be infected by replication-competent HIV-1_IIIB_ or NL4-3 was examined. Human cell lines or PBMCs were used as the positive control, and CRFK or rhesus monkey PBMCs were used as the negative control. Results showed that p24 expression in the culture supernatant of tree shrew cells was almost undetectable, similar to that in the CRFK and rhesus monkey controls (Fig. 1G to J). The expression levels of virus protein p24 in the infected cells and virions produced from cells with the highest multiplicity of infection (MOI) were detected by Western blotting.

Collectively, these results suggest that tree shrew cells efficiently support the postentry steps of HIV-1 infection but lack a functional receptor.

### HIV-1 infection and integration into tree shrew lung fibroblasts expressing exogenous human CD4 and CCR5.

**FIG 2:**
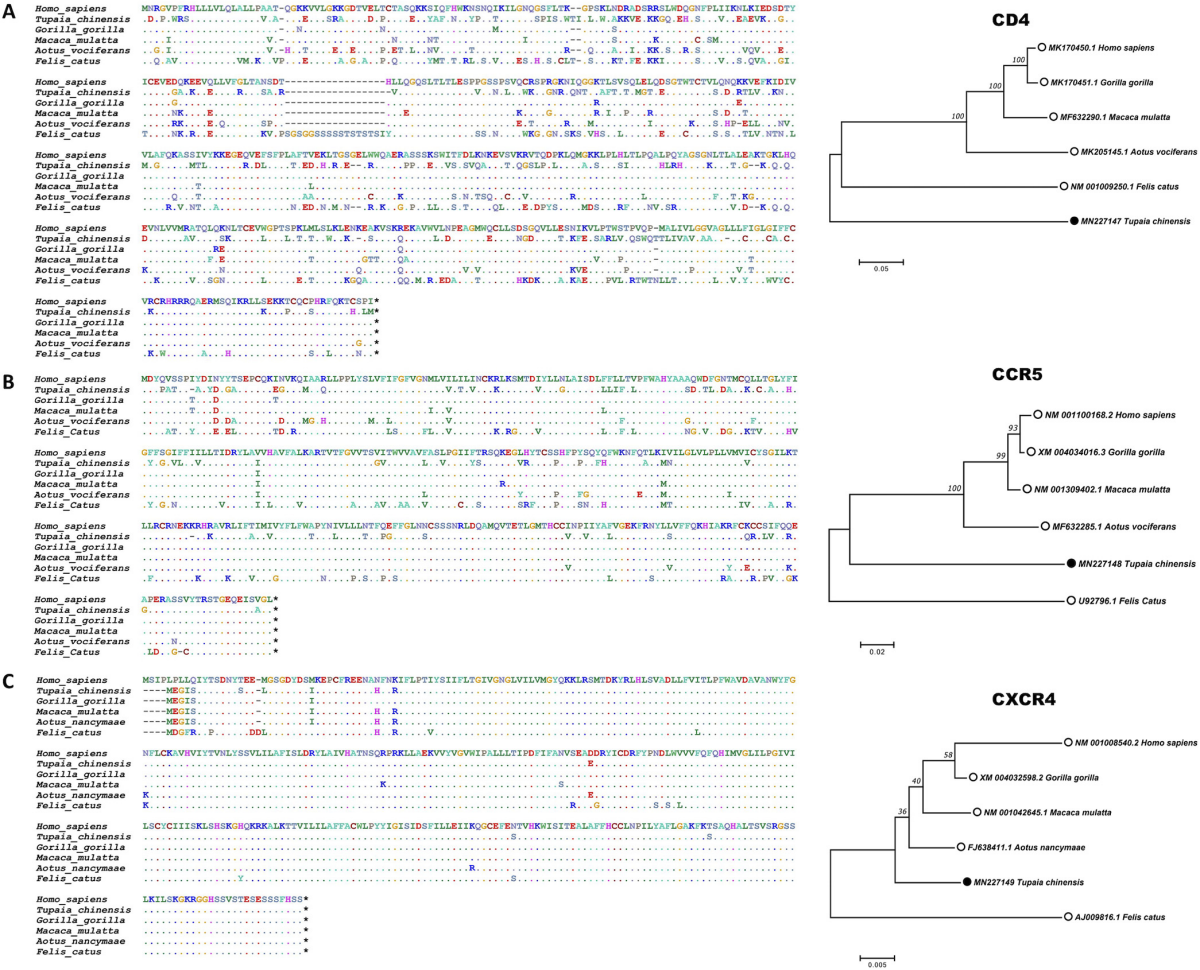
Similarity in CD4, CCR5, and CXCR4 amino acid sequences between humans, macaques, and tree shrews. Tree shrew CD4 (A), CCR5 (B), and CXCR4 (C) amino acid sequences were aligned with analogous sequences. At the amino acid level, homologies between tree shrew and human CD4, CCR5, and CXCR4 were 61.0%, 78.1%, and 97.7%, respectively. Homologies between tree shrew and Gorilla gorilla, Macaca mulatta, Aotus vociferans, and Felis catus CD4 were 56.5%, 55.7%, 54.9%, and 55.5%, respectively; Gorilla gorilla, Macaca mulatta, Aotus vociferans, and Felis catus CCR5 were 78.7%, 79.0%, 78.4%, and 74.7%, respectively; and Gorilla gorilla, Macaca mulatta, Aotus nancymaae, and Felis catus CXCR4 were 96.6%, 96.6%, 96.9%, and 93.6%, respectively. Accession numbers are in front of species name in phylogenetic trees.

The CD4 receptor protein and CCR5 and CXCR4 coreceptors are critical for HIV-1 infection and act as barriers to restrict HIV-1 adhesion and entry into target cells. Research has shown that macaque CD4 supports HIV-1 entry but mouse CD4 does not (8, 42). To examine whether HIV-1 receptors are barriers to cross-species transmission in tree shrews, we aligned the tree shrew CD4, CCR5, and CXCR4 amino acid sequences with the orthologous sequences in humans and macaques. As shown in Fig. 2, the homologies between tree shrew and human CD4, CCR5, and CXCR4 were 61.0%, 78.1%, and 97.7%, respectively (aligned by ClustalW; https://www.genome.jp/tools-bin/clustalw), suggesting that CD4 and CCR5 may be limiting factors for HIV-1 infection in tree shrew cells.

**FIG 3:**
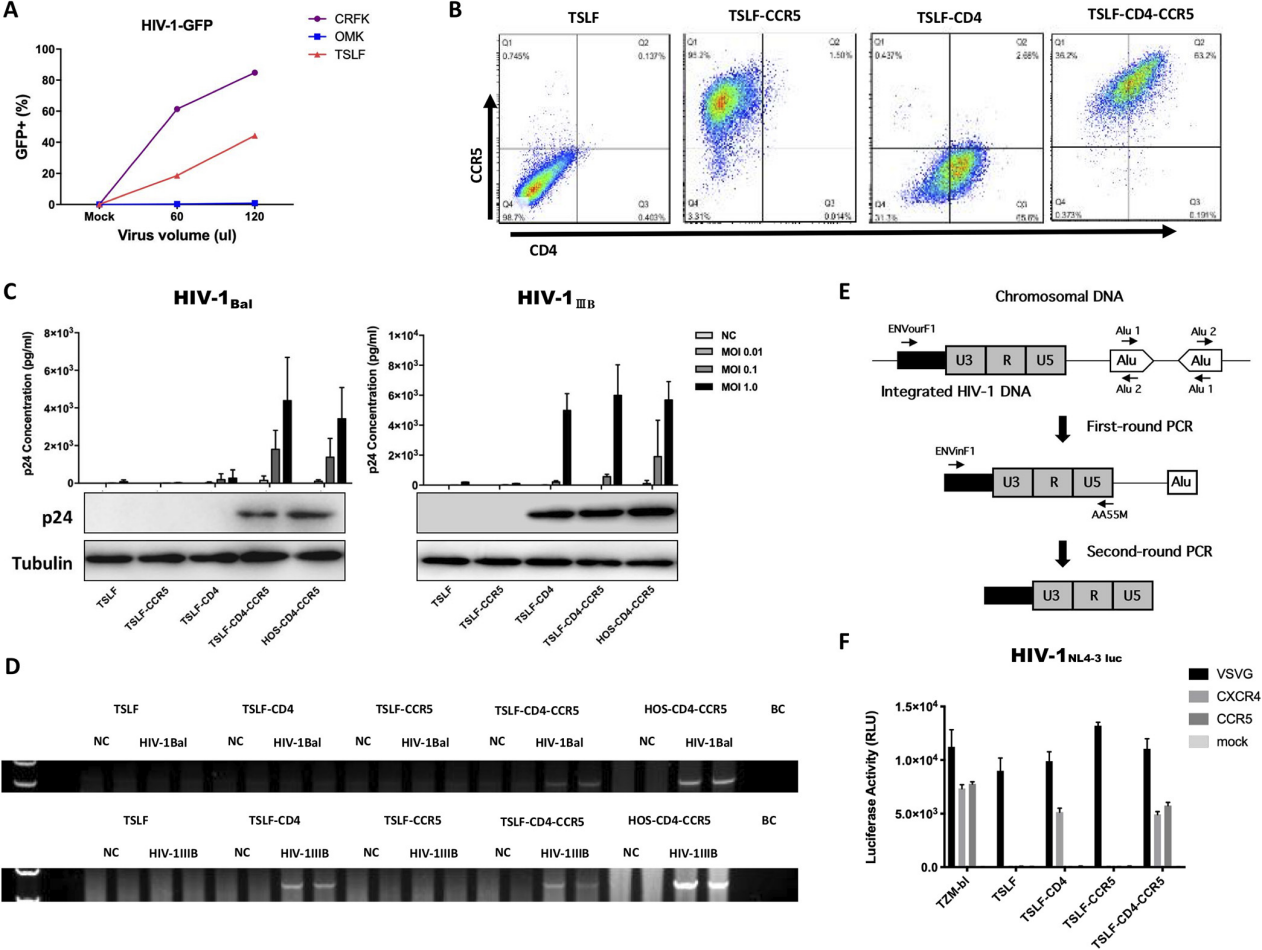
HIV-1 infection and integration in stably transduced TSLF-CD4-CCR5 cell line. (A) CRFK, OMK, and TSLF cells were subjected to infection with various doses (i.e., 0, 60, and 120 μl) of single-cycle VSV-G-pseudotyped VSV-G HIV-1. Percentages of GFP-positive cells were determined at 3 dpi. (B) Expression of human CD4 and CCR5 in TSLFs stably transduced by retroviral expression vectors. Cells were stained for human CD4 with CD4-PE-Cy7 and human CCR5 with CCR5-APC. Fluorescence intensity was analyzed by flow cytometry. (C) TSLF series cell line and HOS-CD4-CCR5 cells were infected with R5-tropic HIV-1_Bal_ and X4-tropic HIV-1_IIIB_ at various MOIs, and p24 expression in the supernatant was analyzed at 3 dpi. Expression levels of p24 in cells at MOI values of 1.0 were analyzed by Western blotting. (D) Integrated HIV-1 DNA was detected in genomes of cells by nested PCR. (E) Integrated HIV-1 DNA detection was modified from the assay to quantify integrated HIV-1 DNA using an *Alu*-long terminal repeat (LTR)-based real-time nested PCR strategy (65). (F) TSLF and TZM-bl cells were infected with HIV-1-luc with VSV-G, CXCR4, or CCR5 envelopes.

To reconstruct tree shrew cell lines that express functional receptors for HIV-1, we first obtained TSLFs. We confirmed whether TSLFs could support the early phases of HIV-1 replication by determining the percentage of GFP-positive cells after single-cycle VSV-G-pseudotyped HIV-1-GFP virus infection (Fig. 3A). We then stably transduced TSLFs with retroviral vectors expressing human CD4 and CCR5. Three cell lines were generated, including single receptor-bearing TSLF-CD4 and TSLF-CCR5 and dual receptor-bearing TSLF-CD4-CCR5 cells. Flow cytometry confirmed the cell surface expression of human CD4 and CCR5 in the TSLF, TSLF-CD4, TSLF-CCR5, and TSLF-CD4-CCR5 cells (Fig. 3B).

Engineered TSLF cell lines were then infected with R5-tropic HIV-1_Bal_ and X4-tropic HIV-1_IIIB_, respectively. Human osteosarcoma (HOS) cells expressing human CD4 and CCR5 (HOS-CD4-CCR5) were used as a positive control. Results showed that the TSLF-CD4-CCR5 cells exhibited p24 levels as high as those found in human HOS-CD4-CCR5 cells with R5-tropic HIV-1_Bal_ infection, whereas the human CD4- and human CCR5-negative tree shrew cells did not allow infection of R5-tropic envelopes (HIV-1_Bal_) (Fig. 3C). The HOS-CD4-CCR5 cells supported X4-tropic HIV-1_IIIB_ infection, as they expressed endogenously functional CXCR4. Remarkably, the TSLF-CD4-CCR5 and TSLF-CD4 cells exhibited p24 levels as high as those found in human HOS-CD4-CCR5 cells infected with X4-tropic HIV-1_IIIB_ (Fig. 3C). Consistently, integrated HIV-1 DNA could be detected in the genome of successfully infected target cells (Fig. 3D) with the method diagram shown in Fig. 3E. Additionally, the use of HIV-1_NL4-3-Luc_-pseudotyped virus with different envelopes in the cell lines yielded similar results, as shown in Fig. 3F. These data indicate that tree shrew cells transduced with human CD4 and CCR5 support HIV-1 entry and replication, suggesting that tsCXCR4 may serve as a functional coreceptor to work with heterogenous human CD4.

### tsCXCR4 is a functional coreceptor for HIV-1 entry.

**FIG 4:**
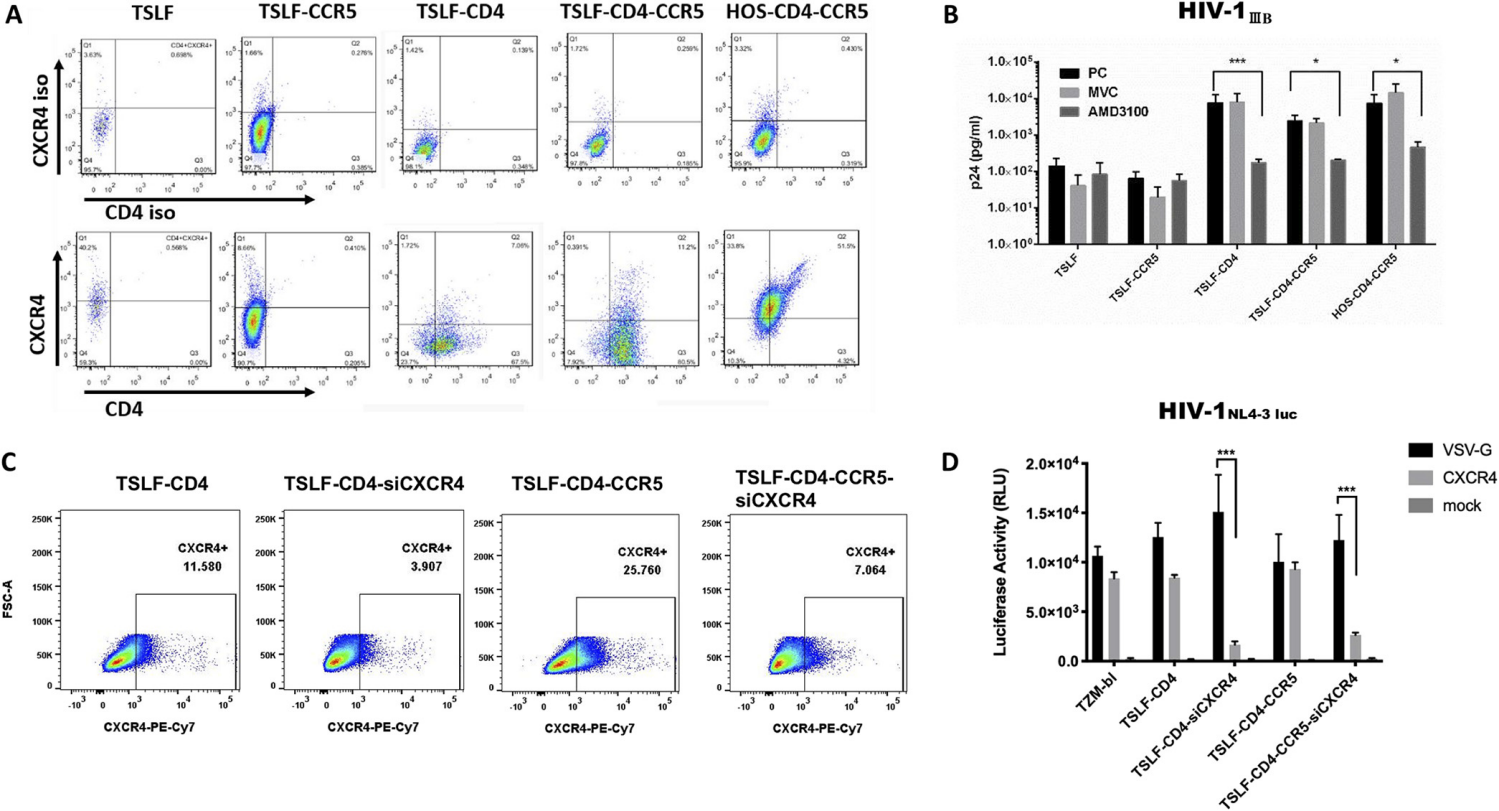
Tree shrew CXCR4 is a coreceptor of HIV-1. (A) Four types of TSLF cells, i.e., TSLF, TSLF-CD4, TSLF-CCR5, and TSLF-CD4-CCR5, and HOS-CD4-CCR5 were detected by flow cytometry with anti-human CXCR4 monoclonal antibody CD184-FITC to examine CXCR4 expression in tree shrew lung fibroblasts. Cells were stained for human CD4 with CD4-PE-Cy7. CXCR4 and CD4 isotype antibodies were used as controls. (B) CXCR4 inhibitor AMD3100 or CCR5 inhibitor MVC were added to cells 2 h before infection. Expression levels of p24 in the supernatant were detected at 3 dpi with MOI of 1.0. (C) Detection of CXCR4 level was performed by flow cytometry with anti-human CXCR4 monoclonal antibody PE-Cy7 to examine CXCR4 expression in CXCR4^kd^ TSLF-CD4 and TSLF-CD4-CCR5 cells. (D) TSLF and TZM-bl cells were infected with VSV-G- or CXCR4-enveloped HIV-1-luc.

We next examined whether tsCXCR4 is functional for HIV-1 entry. First, we performed flow cytometry with an anti-human CXCR4 monoclonal antibody to examine CXCR4 expression in TSLFs. Results showed that four types of TSLFs expressed the CXCR4 chemokine receptor at detectable levels by the anti-human CXCR4 antibody, though the expression levels were lower than that in HOS cells (Fig. 4A). Second, plerixafor (AMD3100), a CXCR4 inhibitor, and maraviroc (MVC), a CCR5 inhibitor, were used as controls (3 μM). Results showed that AMD3100 reversed the X4-tropic HIV-1_IIIB_ infection in permissively engineered tree shrew cells, with the p24 level in the supernatant almost 1,000-fold lower than that in the untreated cells or MVC control (Fig. 4B).

In addition, we knocked down tsCXCR4 expression in TSLFs expressing human CD4 or CD4-CCR5 (Fig. 4C). The CXCR4 envelope HIV-1_NL4-3-Luc_-pseudotyped virus was used for infection. As shown in Fig. 4D, the luciferase signal was significantly lower in the tsCXCR4 knockdown cells than in the control cells. These findings indicate that tsCXCR4 is a coreceptor for HIV-1 adhesion and entry.

### Impaired viral infectivity of HIV-1 progeny from TSLF-CD4-CCR5 cells.

**FIG 5:**
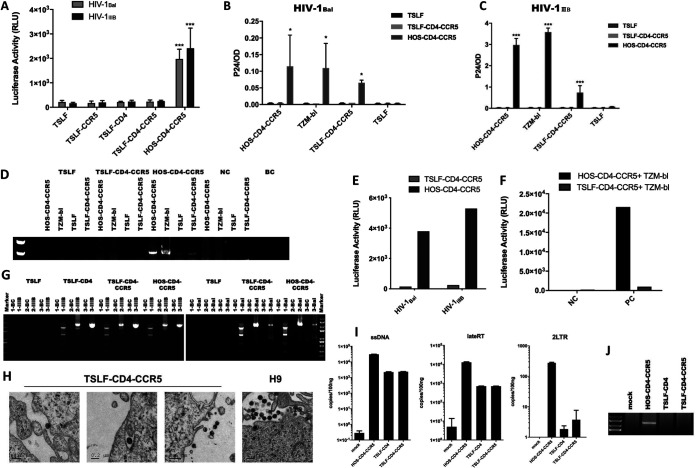
Impaired viral infectivity of HIV-1 progeny from TSLF-CD4-CCR5 cells. (A) Various tree shrew TSLF cell lines were infected at an MOI of 1.0 with replication-competent HIV-1_Bal_ and HIV-1_IIIB_; at 3 dpi, the supernatant was normalized to 0.3 ng of p24 to infect TZM-bl reporter cells. After 3 days, luciferase activity was detected. (B and C) HOS-CD4-CCR5 and TSLF-CD4-CCR5 cells were infected at an MOI of 1.0 with replication-competent HIV-1_Bal_ (B) and HIV-1_IIIB_ (C); at 3 dpi, the supernatant normalized to 0.3 ng of p24 was used to reinfect HOS-CD4-CCR5, TSLF-CD4-CCR5, TSLF, and TZM-bl cells. At 3 dpi, p24 expression was detected by ELISA. (D) Integrated HIV-1 DNA was detected in the genome of second-round target cells by nested PCR. (E) Tree shrew TSLF and HOS-CD4-CCR5 cells were infected with replication-competent HIV-1_IIIB_; at 3 dpi, the supernatant was normalized to 0.8 ng of p24-equivalent virus to reinfect cells. (F) TSLF-CD4-CCR5 cells infected with HIV-1_IIIB_. At 24 h, after washing free virus, TSLF-CD4-CCR5 cells were cocultured with TZM-bl to mimic cell-to-cell infection, with luciferase activity detected at 3 dpi. NC, no virus; PC, HIV-1_IIIB_. (G) RNA from culture supernatant was extracted and amplified; progeny virus full-length genome was divided into three sections. Target sections were detected by agarose gel electrophoresis and sequenced. Fragment 1 was 2.5 kb, and fragments 2 and 3 were 3.5 kb. (H) TSLF-CD4-CCR5 cells were infected at an MOI of 1.0 with replication-competent HIV-1_IIIB_. At 3 dpi, viral particles were examined by electron microscopy (three images on left). Viral particles in H9 cells were used as controls (right). (I) Progeny viruses from TSLF-CD4-CCR5 and HOS-CD4-CCR5 cells were used to reinfect C8166 cells. After 4, 8, and 24 h, whole DNA was extracted, and virus products of ssDNA, lateRT, and 2LTR were determined by RT-qPCR. (J) Integrated HIV-1 DNA was detected in the genome of C8166 cells by nested PCR at 36 hpi. Supernatant from TSLF cells, which could not be infected by HIV-1_IIIB_, is marked as “mock.”

We next evaluated the infectivity of the progeny virus produced from the receptor-engineered tree shrew cells. A high multiplicity of infection (MOI) (1.0) of replication-competent HIV-1_Bal_ or HIV-1_IIIB_ was used to infect target tree shrew cells. At 48 h postinfection (hpi), the cell culture supernatant was normalized to 0.3 ng of p24 for the infection of TZM-bl reporter cells. We showed that luciferase activity in the TZM-bl cells infected with the supernatant from the receptor-engineered tree shrew cells was close to that of the negative control infected with the TSLF supernatant but much lower than that of TZM-bl cells infected with the HOS-CD4-CCR5 supernatant. Thus, the progeny viruses from the tree shrew TSLF-CD4 and TSLF-CD4-CCR5 cells were likely deficient for reinfection (Fig. 5A). To exclude the possibility that cell species may affect progeny virus infectivity, we infected other cells with 0.3 ng of p24 from the culture supernatant from the first-round infection. As shown in Fig. 5B to D, only the progeny virus from HOS-CD4-CCR5 cells could reinfect HOS-CD4-CCR5, TSLF-CD4-CCR5, and TZM-bl cells, showing detectable p24 levels in the supernatant and integrated proviral DNA in the cells, whereas viruses produced from the TSLF or TSLF-CD4-CCR5 cells were noninfectious, even when a larger input load of progeny virus was used to reinfect the cells (Fig. 5E). In addition, in the cell-to-cell infection assay, luciferase activity was undetectable in TSLF-CD4-CCR5 cells cocultured with TZM-bl but not in HOS-CD4-CCR5 cells cocultured with TZM-bl (Fig. 5F).

With the observation that tree shrew cells were permissive to the VSV-G HIV-1-GFP reporter virus but produced low-infectivity virions, we next investigated the underlying molecular basis. We first characterized the progeny virus genome from the infected tree shrew cells. We extracted genomic RNA from the culture supernatant and then amplified the full-length genome. As detected by agarose gel electrophoresis, there were no obvious differences between the progeny virus genome from the HOS-CD4-CCR5 cells and the TSLF-CD4-CCR5 cells (Fig. 5G). Additionally, there was no severe mutation in the progeny virus genome sequence from the TSLF-CD4-CCR5 cells (data not shown). The progeny virus from the TSLF-CD4-CCR5 cells was as morphologically normal as that from the chronically infected H9 cells and could enter target cells successfully (Fig. 5H). However, we found that the viral products of early and late reverse transcription, as well as the products of viral nucleus transport, were impaired in C8166 cells infected by the progeny virus from TSLF-CD4-CCR5 compared to C8166 cells infected by the progeny virus from HOS-CD4-CCR5 (Fig. 5I). Furthermore, the subsequent integration process was not detectable (Fig. 5J). Overall, these findings showed that the infectivity of the progeny viruses produced from the receptor-engineered tree shrew cells was greatly impaired.

### tsAPOBEC3 proteins inhibit infectivity of HIV-1 progeny.

**FIG 6:**
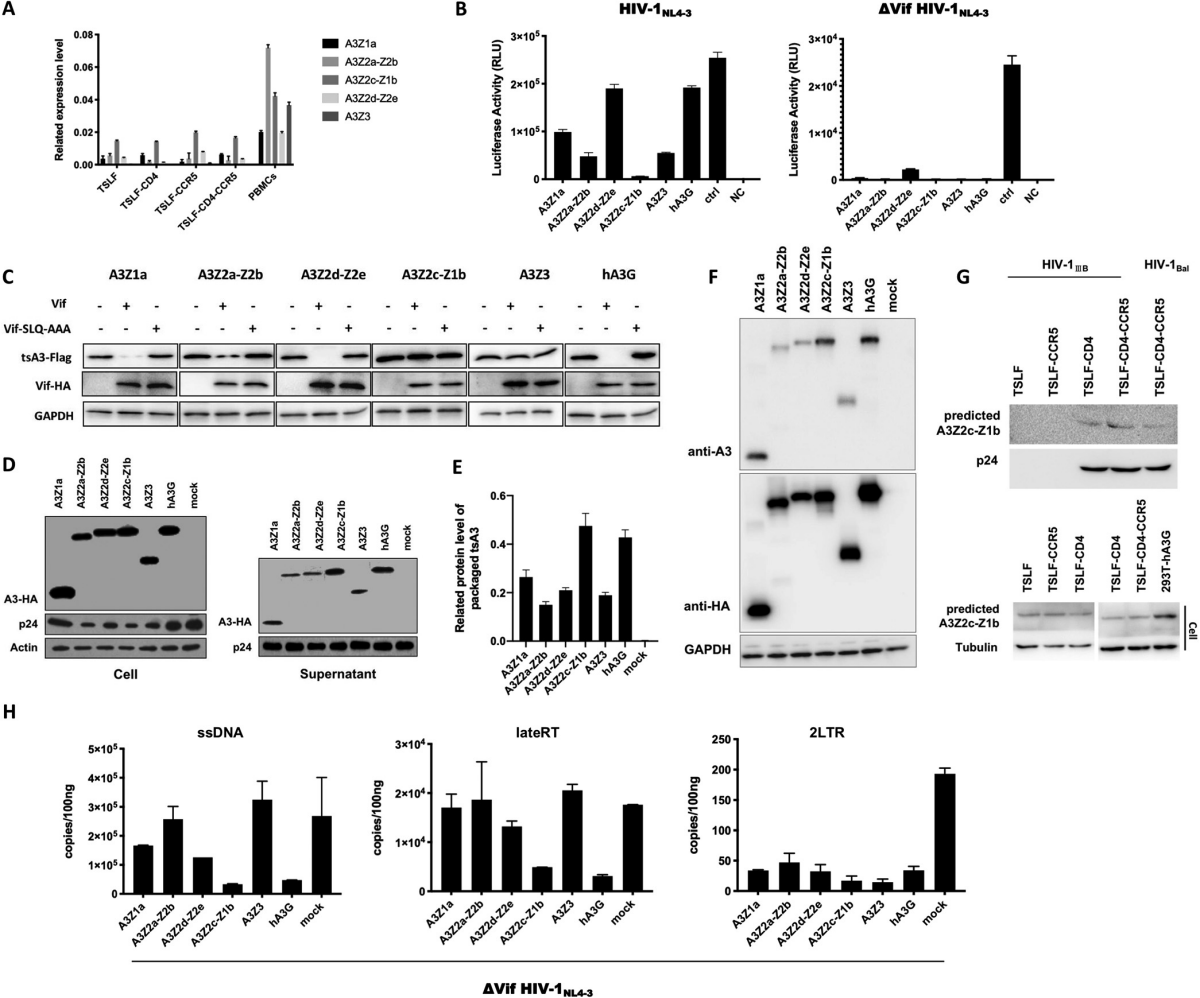
Tree shrew APOBEC3s restricted HIV-1 replication. (A) APOBEC3 mRNA levels in TSLF cells and tree shrew PBMCs were detected by RT-qPCR. (B) Wild-type pNL4-3 or Vif-deficient pNL4-3 and tsAPOBEC3 expression plasmids were cotransfected at 5:1 into human 293T cells. Infectivity of progeny viruses was determined by quantifying luciferase activity in TZM-bl cells with equal amounts of 5 ng of p24. Control, no A3 protein; NC, no A3 protein, no virus. (C) tsAPOBEC3 or huA3G, and Vif or Vif-SLQ-AAA expression plasmids were cotransfected at 1:5 into 293T cells. Immunoblot analysis results of HIV-1 Vif protein and tsAPOBEC3 protein expression are shown. tsA3Z2c-Z1b and tsA3Z3 were not degraded by Vif. (D) The delta Vif HIV-1_NL4-3_ and tsAPOBEC3 expression plasmids were cotransfected at 5:1 into 293T cells. At 3 dpi, cells or virions from supernatants were analyzed by immunoblots using p24 and HA-specific monoclonal antibody. Mock, empty pcDNA3.1 vector. (E) Gray analysis of tsAPOBEC3 proteins packaged in viral particles. (F) tsAPOBEC3 proteins were detected by mouse anti-hAPOBEC3G antibody. (G) At 3 dpi, virions in supernatants from HIV-1-infected TSLF series cells were analyzed on immunoblots using p24 and hAPOBEC3G-specific monoclonal antibody. TSLF series cells were analyzed on immunoblots using hAPOBEC3G monoclonal antibody. The overexpression of hAPOBEC3G protein in 293T cells was used as a positive control. (H) Progeny viruses were used to synchronously reinfect C8166 cells. After 4, 8, and 24 h, whole DNA was extracted, and ssDNA, lateRT, and 2LTR were determined by RT-qPCR. Mock, no A3.

The above results suggest that tree shrew cells may express certain restriction factors that affect the production of infectious HIV-1 progeny virus. It has been reported that human APOBEC3G (huA3G) can be packaged into viral particles and induce G-to-A hypermutations in the viral genome during reverse transcription in the target cells (43, 44). To counteract huA3G, HIV-1 produces a protein called Vif to degrade huA3G and therefore exclude huA3G packaging in the virions (45, 46). We previously identified five tsAPOBEC3 member proteins that could induce G-to-A and C-to-T hypermutations in the HIV-1 genome (35). Based on this observation, we wondered if tsAPOBEC3 proteins play a part in the restriction of HIV-1. In the TSLF-CD4-CCR5 HIV-1 infection system, we found that tsAPOBEC3 proteins were expressed in TSLF cells (Fig. 6A).

To assess the antiviral activity of tsAPOBEC3 proteins, wild-type HIV-1_NL4-3_, Vif-deficient HIV-1_NL4-3_, and tsAPOBEC3 plasmids or positive-control huA3G were cotransfected into human 293T cells, with equal amounts of produced p24 then used for reinfection in TZM-bl reporter cells. As reported previously, human APOBEC3G reduces infectivity of Vif-defective HIV-1 but not of wild-type HIV-1 (45, 47, 48). Likewise, all five tsAPOBEC3s reduced the infectivity of Vif-defective HIV-1 by 10- to 100-fold. Surprisingly, four out of the five tsAPOBEC3 proteins were strong suppressors of wild-type HIV-1 infectivity, particularly tsA3Z2c-Z1b (Fig. 6B), suggesting that the anti-HIV-1 activity of tsA3Z2c-Z1b was not counteracted by HIV-1 Vif. To determine whether tsAPOBEC3 could be degraded by HIV-1 Vif, we transfected tsAPOBEC3, huA3G, and Vif or Vif-SLQ-AAA expression plasmids into 293T cells (Vif-SLQ-AAA impairs Vif-EloB-EloC binding [49]). As shown in Fig. 6C, HIV-1 Vif markedly degraded huA3G, as reported previously (45, 50, 51), and degraded tsA3Z1a, tsA3Z2a-Z2b, and tsA3Z2d-Z2e to a certain extent but did not degrade tsA3Z2c-Z1b or tsA3Z3. In addition, we found that tsAPOBEC3 proteins could be packaged into HIV-1 particles, the same as huA3G (Fig. 6D). Thus, tsA3Z2c-Z1b may be efficiently encapsulated into viral particles (Fig. 6E). Due to the lack of tsAPOBEC3-specific antibodies, we verified the hAPOBEC3G antibodies could predict the tsAPOBEC3 proteins first (Fig. 6F). In the TSLF-CD4-CCR5 HIV-1 infection system, then we predicted the presence of tsA3Z2c-Z1b or tsA3Z2d-Z2e proteins in viral particles from the culture supernatant by hAPOBEC3G antibodies based on molecular weight (Fig. 6G).

As the suppression of reporter proteins is correlated with a significant increase in G-to-A hypermutations in viral reverse transcription products or with restricted elongation of reverse transcripts in target cells (52), we detected whether the viral products were affected by tsAPOBEC3 proteins. Progeny viruses produced from 293T cells cotransfected with tsAPOBEC3 and pNL4-3-Δvif plasmids were first used to infect C8166 cells. Whole DNA of the C8166 cells was then extracted for the detection of ssDNA, completion of reverse transcription (lateRT), and transportation to the nucleus (2LTR) by RT-qPCR. Results showed that tsA3Z2c-Z1b strongly inhibited reverse transcription, late reverse transcript production, and nuclear transport (Fig. 6H).

### Depletion of tsA3Z2c-Z1b increases infectivity of progeny virus from tree shrew cells.

**FIG 7:**
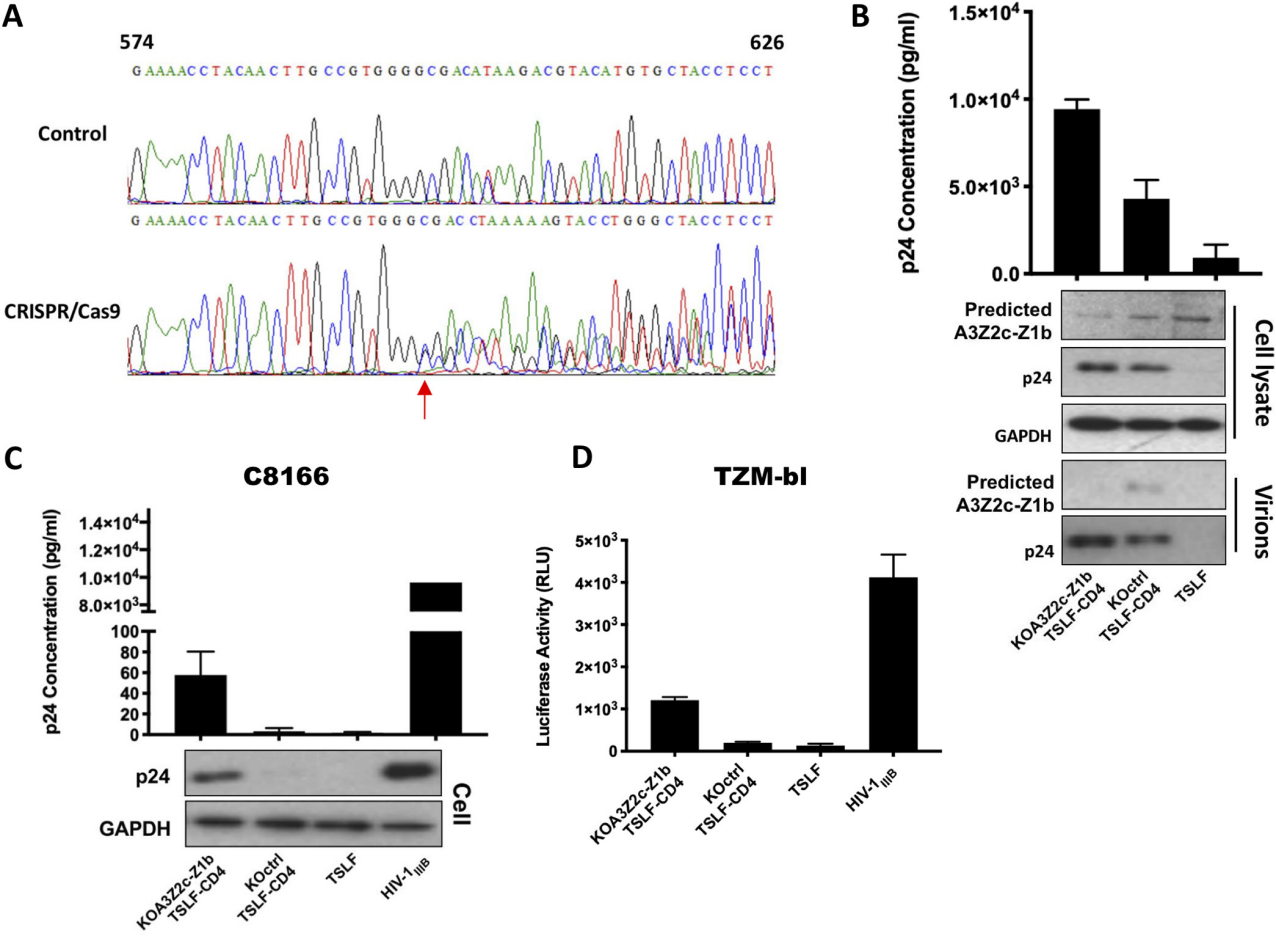
Depletion of tsA3Z2c-Z1b increased infectivity of progeny virus from tree shrew cells. (A) tsA3Z2c-Z1b genome sequences in TSLF-CD4 and tsA3Z2c-Z1b-KO mixed TSLF-CD4 cells were detected by PCR and then sequenced. Arrow indicates beginning of mutation. (B) tsA3Z2c-Z1b-knockout TSLF-CD4, TSLF-CD4, and TSLF cell lines were infected at an MOI of 2.0 with replication-competent HIV-1_IIIB_. Expression levels of p24 were detected by ELISA at 3 dpi. tsA3Z2c-Z1b and virus p24 protein from cells and supernatants were detected by Western blotting. (C) At 3 dpi, supernatant from panel B was normalized to 1 ng of p24 to infect C8166 cells. p24 expression was detected by ELISA at 3 dpi. (D) At 3 dpi, supernatant from panel B was normalized to 10 ng of p24 to infect TZM-bl cells. After 3 days, luciferase activity was detected. Positive-control virus HIV-1_IIIB_ was made in C8166 cells.

The above findings showed that tsA3Z2c-Z1b has strong anti-HIV activity when overexpressed. To explore the effects of tsA3Z2c-Z1b on the infectivity of progeny virus *in vitro*, we generated a pooled tsA3Z2c-Z1b-knockout (KO) TSLF-CD4 cell line using CRISPR/Cas9. The tsA3Z2c-Z1b genome was sequenced in the tsA3Z2c-Z1b-KO mixed TSLF-CD4 cells. The tsA3Z2c-Z1b gene was edited to form a frameshift mutation using CRISPR/Cas9 (Fig. 7A). As shown in Fig. 7B, when the predicted endogenous expression of tsA3Z2c-Z1b was efficiently reduced as indicated by Western blotting, p24 production was higher than that in the knockout control from the first round of HIV-1_IIIB_ infection. The progeny virus was then used to reinfect C8166 and TZM-bl cells with normalized 1 ng or 10 ng of p24, respectively. The progeny virus from the tsA3Z2c-Z1b-knockout TSLF-CD4 cells effectively reinfected the C8166 and TZM-bl cells, whereas the progeny virus from the TSLF-CD4 cells lacked infectivity (Fig. 7C and D). Thus, the depletion of endogenous tsA3Z2c-Z1b helped to restore the infectivity of the HIV-1 progeny virus, demonstrating its critical role in HIV-1 restriction.

In conclusion, the tsAPOBEC3 proteins, especially tsA3Z2c-Z1b, play an important role in suppressing the infectivity of HIV-1 virions and significantly contribute to the blocking of HIV-1 propagation in TSLF-CD4-CCR5 cells.

## DISCUSSION

In this study, we characterized the ability of tree shrew cells to support HIV-1 replication to determine whether tree shrews can be developed into animal models for HIV-1 studies. We found that the exogenous expression of human CD4 and CCR5 molecules in tree shrew cells was sufficient for HIV-1 entry and replication and that tsCXCR4 serves as a functional coreceptor. However, additional blocks to HIV-1 infection were identified in the tree shrew cells. In particular, tree shrew cytidine deaminases of the APOBEC3 family were found to exert a strong inhibitory effect on HIV-1 replication.

Since the discovery of HIV in 1983 as the pathogen of AIDS, our understanding of the virus has greatly improved. However, the development of effective animal models is essential for continued study of the pathogenesis of AIDS and to evaluate the efficacy of drugs and vaccines. The ideal animal model of HIV infection should be a small laboratory mammal that closely mimics virus replication in humans. However, suitable small animal models of HIV-1 do not yet exist, though a variety of feline and humanized rodent models of HIV-1 infection have been developed (1, 8, 9, 53–55). The failure of establishing HIV-1 models is primarily related to functional receptors or coreceptors and postinfection host restriction factors. A lack of functional receptors is the first impediment to HIV-1 infection in murine, feline, and ferret cells (1, 8, 56). Restriction factors, on the other hand, constitute the main impediment of HIV-1 replication in simian cells. For example, cytoplasmic-body protein TRIM5α restricts HIV-1 replication at the postentry step in simian cells (57). In contrast, the TRIMCyp fusion protein expressed in pig-tailed macaques exhibits no restriction to HIV-1 activity, identifying a potential molecular mechanism that may explain why pig-tailed macaques are prone to HIV-1 infection (58). Murine and feline cells exhibit additional restrictive factors for HIV-1 replication other than setting receptor limitations: murine cells lack LTR-driven transcription functions (8, 55), and feline APOBEC3 proteins restrict HIV-1 replication by inducing G-to-A hypermutation in the viral genome (1).

We previously showed that tree shrews express APOBEC3 proteins that induce G-to-A and C-to-T hypermutations in the HIV-1 genome (35). In the tree shrew hepatitis B virus (HBV) model, tsAPOBEC3 may limit the establishment of HBV-persistent infection due to its strong anti-HBV replication ability (59). Here, we investigated whether tsAPOBEC3 proteins can account for the failure of tree shrew cells engineered with human CD4 and CCR5 receptors to produce infectious particles. The APOBEC3 protein is usually packaged into retroviral particles from the producing cells and is transmitted to the target cells through infection (26). “Nonpermissive” cells exhibit APOBEC3G expression and are resistant to delta Vif HIV-1 replication. To antagonize the restriction of APOBEC3G, HIV-1 encodes the Vif to degrade human APOBEC3 proteins and reduce the incorporation of APOBEC3 into progeny virions (60). HIV-1 is strongly inhibited by simian APOBEC3G and murine APOBEC3 because the charge of amino acid 128 determines its functional interaction with Vif. When this site is positively charged, HIV-1 Vif cannot degrade APOBEC3G (61–63). In this context, the fact that tsA3Z2c-Z1b was potently restrictive to HIV-1 and not degraded by the Vif may be the cause of the low infectivity of the progeny virus from TSLF-CD4-CCR5, making TSLF-CD4-CCR5 cells nonpermissive and therefore potentially inappropriate as an HIV-1 cell model.

In summary, we observed multiple restrictions against HIV-1 replication in tree shrew cells in this study. We characterized tree shrew membrane receptors and APOBEC3 proteins as limitations that initiate the restrictions. Further detailed analysis of why HIV-1 cannot overcome the cellular barriers of tree shrews or other animals will likely generate knowledge of how to genetically modify human cells against HIV-1 replication and help to better understand how to prevent viral cross-species transmission.

## MATERIALS AND METHODS

### Collection of tree shrew tissues and isolation of cells.

Tree shrews were raised at the Experimental Core Facility of the Kunming Institute of Zoology, Chinese Academy of Sciences. Brain, heart, liver, lung, spleen, colon, and blood samples were isolated from healthy male adult and newborn tree shrews. All experimental procedures were performed according to the guidelines approved by the Ethics Committee of the Kunming Institute of Zoology (approval number SYDW-2015017).

### Cell culture.

Human PBMCs (huPBMCs) were activated with phytohemagglutinin (PHA; Sigma, USA) and 10 U/ml interleukin-2 (IL-2), while tree shrew (tsPBMCs) and rhesus macaque PBMCs (rhPBMCs) were activated with concanavalin A (ConA; Sigma, USA). After 72 h of activation, the PBMCs were grown in RPMI 1640 medium supplemented with 10% fetal bovine serum (FBS) and 10 U/ml IL-2.

Primary tissue cultures of newborn and adult tree shrews were established by mincing dissected organs, followed by trypsinization for 30 min at 37°C. Cells were washed, filtered through a 40-cm pore tissue culture mesh filter (Falcon), and then grown in Dulbecco’s modified Eagle’s medium (DMEM) with 10% FBS and penicillin and streptomycin.

Cell lines, including CRFK, OMK, 293T, TZM-bl, HOS-CD4-CCR5, and TSLF or TSLF subset cells, were cultured in DMEM supplemented with 10% FBS. C8166 cells were cultured in RPMI 1640 supplemented with 10% FBS.

### Plasmids and transfection.

The pcDNA3.1-tsAPOBEC3 plasmids were generated by inserting tsAPOBEC3 cDNA (GenBank accession nos. KU053484, KU053485, KU053486, KU053487, and KU053488) into pcDNA3.1(+) script (Invitrogen) fused to the hemagglutinin (HA) or Flag epitope tag at its amino terminus. pLVX-human-CD4-IRES-Neo was generated by inserting human CD4 (GenBank accession no. MK170450.1) into pLVX-IRES-Neo (8,316 bp), and pCDH-EF1-human-CCR5-T2A-Puro was generated by inserting human CCR5 (GenBank accession no. AY463214.1) into pCDH-EF1-MCS-T2A-Puro. The pNL4-3 plasmid and 293T and TZM-bl cell lines were obtained from the NIH AIDS Reagent Program. HIV-1ΔVifNL4-3 was a kind gift from Yong-Hui Zheng (Michigan State University, USA). The pcDNA3.1-Vif-HA plasmid was kindly donated by Hui Zhang (Institutes of Human Virology, Sun Yat-sen University, China), and the pcDNA3.1-Vif-SLQ-AAA-HA SLQ mutation plasmid was generated by point mutation. PX330-mCherry as a single guide RNA (sgRNA)-Cas9 vector was generated to knock out tsA3Z2c-Z1b following a previous study (64). The sgRNA design was achieved using the online tool Breaking-Cas (https://bioinfogp.cnb.csic.es/tools/breakingcas/index.php). The tsA3Z2c-Z1b sgRNA-F primer was 5′-CACCGGTACGTCTTGTGTCGCCCCA-3′, and sgRNA-R was 5′-AAACTGGGGCGACACAAGACGTACC-3′. The KO-checking primers were check-F, 5′-ATCTTCCTTTTCCCTCTGACCTTCAGGCC-3′, and check-R, 5′-CGTCCAGCAGCATGAACGTGGCCC-3′.

Lentiviruses were produced in 293T cells by cotransfecting psPAX2, pMD.2G, and pLVX-human-CD4-IRES-Neo or pCDH-EF1-human-CCR5-T2A-Puro. The TSLF-CD4-CCR4 cells were generated by transduction with lentiviral vectors and then cultured in medium containing G418 and puromycin. The 293T cells were cotransfected with HIV-1 NL4-3 plasmids and tsAPOBEC3 with Lipofectamine 2000 (Invitrogen, USA). Levels of p24 were detected in the supernatant 48 to 72 h later. The tsA3Z2c-Z1b-PX330-mCherry was transfected into the TSLF-CD4 cells with Lipofectamine LTX (Invitrogen, USA). Positive cells were sorted by flow cytometry 48 h later and verified by PCR.

### Virus production.

Single-cycle VSV-G-pseudotyped viruses were produced by transfection of 293T cells using Lipofectamine 2000. HIV-1-GFP and SIVmac-GFP were produced with pCMV-dR8.2 and pCMV-VSV-G, as described previously (39). Both pCMV-VSV-G and pNL4-3R-E- were cotransfected into 293T cells at a ratio of 1:4 to generate single-cycle VSV-G-pseudotyped HIV-1-GFP, while pCMV-VSV-G and pNL4-3 were cotransfected into 293T cells at a ratio of 1:5 to generate replication-competent VSV-G HIV-1_NL4-3_. pNL4-3 R-E-Luc is an HIV-1_NL4-3_ plasmid with a luciferase reporter gene and no REV envelope. Here, pNL4-3-Luc and HXB2, JRFL, or pCMV-VSV-G were cotransfected into 293T cells at a ratio of 1:4 to generate X4, R5, or VSV-G envelope-pseudotyped HIV-1_NL4-3-Luc_. Furthermore, pLKO.1-shtsCXCR4, psPAX2, and pMD2.G were cotransfected into 293T cells at a ratio of 4:3:1 to generate tsCXCR4-knockdown lentiviral particles.

### Infection assays.

For single-cycle infection assays, cells were seeded in 24-well plates at a density of 5 × 10^4^ cells/well and incubated overnight at 37°C in 5% CO_2_. The cells were then infected with VSV-G-pseudotyped viruses. At 48 hpi, cells were washed with phosphate-buffered saline (PBS), and then the percentage of GFP-positive cells was examined by flow cytometry.

For the PBMC infection assays, freshly activated PBMCs (5 × 10^6^ cells) were seeded in 24-well plates and infected with HIV-1. At 16 hpi, cells were washed three times with PBS and resuspended in fresh RPMI-10% FBS supplemented with IL-2. Supernatants were collected at 72 hpi. The number of viral particles released into the cell culture supernatant was quantitated by p24 enzyme-linked immunosorbent assay (ELISA; ZeptoMetrix, USA).

For multiple-cycle infection assays, cells were seeded in 12-well plates incubated overnight at 37°C in 5% CO_2_. The cells were then infected with HIV-1 or transfected with HIV-1 expression plasmids, with the culture medium refreshed 6 h later. If an inhibitor was needed, 100 nM IDV or 3 μM AMD3100/MVC was added. IDV was added as a control to detect the infection system. Plerixafor (AMD3100) is a CXCR4 inhibitor, and MVC is a CCR5 inhibitor. After 48 to 72 h, the supernatants and cells were collected to detect p24 by ELISA and virus/cell proteins by Western blot analysis. The supernatants were normalized to the same viral level for reinfecting target cells. After 72 h, the cells or culture supernatants were collected to detect luciferase activity or p24 levels.

For HIV-1_NL4-3-Luc_ infection assays, cells were seeded in 24-well plates at a density of 5 × 10^4^ cells/well and incubated overnight at 37°C in 5% CO_2_. The cells were then infected with HIV-1_NL4-3-Luc_ viruses. At 72 hpi, cells were washed with PBS and then collected to detect luciferase activity.

### Flow cytometry.

TSLF cells were stained with CD4-PE-Cy7 (BD, USA), CCR5-APC (BioLegend, USA), and CD184-fluorescein isothiocyanate (FITC) (BioLegend, USA) to detect CD4, CCR5, and CXCR4 expression, respectively. CXCR4-PECy7 (BioLegend, USA) was used to detect CXCR4 knockdown. The percentage of GFP-positive cells was examined on a BD Biosciences FACSVerse flow cytometer (USA) driven by FACSuite v1.0.3. Analysis of the acquired data was performed using FlowJo v7.6.1 (TreeStar Inc., USA).

In the flow cell-sorting experiment, mCherry-positive cells were sorted using a Sony SH800 flow cytometer (Japan) driven to gain TSLF-CD4 tsA3Z2c-Z1b knockout cells.

### ELISA for quantification of p24 levels.

At 48 to 72 hpi, the cultured supernatants were collected and centrifuged at 200 × *g* for 5 min at room temperature to remove cell debris. The culture supernatants were analyzed using commercial HIV-1 p24 ELISA kits (Wantai BioPharm, China).

### Western blot analysis.

Cells were lysed for 30 min on ice and then centrifuged at 13,000 × *g* for 10 min at 4°C, with the supernatants then transferred into clean 1.5-ml Eppendorf tubes.

The supernatants were centrifuged at 13,000 × *g* for 5 min at room temperature to remove cell fragments 72 h later. After resuspending the precipitation with polyethylene glycol 8000 (PEG 8000) overnight, the supernatants were centrifuged at 13,000 × *g* for 30 min at 4°C to collect viral particles. The particles were lysed with lysis buffer for 30 min on ice and then stored at −80°C.

The prepared proteins were separated by sodium dodecyl sulfate-polyacrylamide gel electrophoresis (SDS-PAGE) and then transferred onto polyvinylidene fluoride (PVDF) membranes (Millipore, USA). The blots were probed with a primary antibody at 4°C and then a secondary antibody at room temperature for 1 h. Afterward, the blots were illuminated with chemiluminescent detection reagents (Millipore, USA).

The serum of HIV-1-positive patients (obtained from Kunming Third People’s Hospital, China) was used to detect glycoprotein 120 (gp120). The mouse anti-p24 polyclonal antibody (produced in-house) was used to detect p24 and p55. The mouse anti-HA monoclonal antibody (catalog no. H3663; Sigma-Aldrich, USA) or anti-Flag monoclonal antibody (catalog no. F1804; Sigma-Aldrich, USA) was used to detect tsAPOBEC3. The mouse hAPOBEC3G monoclonal antibody (ImmunoDiagnostics, USA) was used to detect tsA3Z2c-Z1b.

The other antibodies used were as follows: anti-actin monoclonal antibody (catalog no. cw0096a; CWBIO, China), anti-tubulin monoclonal antibody (Proteintech, USA), anti-GAPDH (glyceraldehyde-3-phosphate dehydrogenase) monoclonal antibody (Proteintech, USA), goat anti-human IgG(H+L) antibody (catalog no. PH0655; Phygene, China), and goat anti-mouse IgG(H+L) antibody (catalog no. 474-1806; KPL, USA).

### Quantitative real-time PCR.

Total DNA from cells was isolated using a TIANamp genomic DNA kit (Tiangen Biotech, Beijing). For the virus genome, commencement of reverse transcription (ssDNA), completion of reverse transcription (lateRT), and transportation to the nucleus (2LTR) were determined by RT-qPCR, as described previously (65).

Total RNA from cells was isolated and then reverse transcribed into cDNA using the PrimeScript RT reagent kit with gDNA Eraser (TaKaRa, Dalian, China). To quantify tsAPOBEC3 mRNA expression levels, RT-qPCR was performed as per previous study (35).

### Provirus HIV-1 DNA detection.

After virus infection, the cells were collected to extract total DNA to detect provirus DNA following earlier research (66). Lentiviral vectors were used to establish stably transfected cells; therefore, we modified the primers to bypass the reverse plasmid skeleton (Fig. 3E). The primers for nested PCR were as follows: (i) 5′-AGA RGA YAG ATG GAA CAA GCC CCA G-3′ (ENVoutF1, first round, forward), 5′-AGA ATG RWG GAG AAG GGA TTG T-3′ (AluI, first round, reverse), or 5′-AGA GAG AGA CAR ASA GAC AGA AAG-3′ (AluII, first round, reverse); (ii) 5′-TGG AAG CAT CCR GGA AGT CAG CCT-3′ (ENVinF1, second round, forward) and 5′-GCT AGA GAT TTT CCA CAC TGA CTA A-3′ (AA55M, second round, reverse).

The following conditions were used for the first round of PCR: 94°C for 8 min; followed by 94°C for 30 s, and 50°C for 45 s; and 72°C for 4 min for 12 cycles and a final extension at 72°C of 10 min. The first-round PCR products were used as a template for the second round of PCR, which was performed with the following conditions: 94°C for 8 min; followed by 94°C for 30 s, 50°C for 45 s, and 72°C for 4 min for 35 cycles; and finally, 10 min at 72°C. The PCR products were detected using agarose gels.

### Full-length HIV-1 RNA detection.

To detect the progeny virus genome from infected tree shrew cells, we extracted genomic RNA from the culture supernatant of HOS-CD4-CCR5 and TSLF-CD4-CCR5 cells and then amplified the full-length genome. The full-length genome was divided into three sections for detection. The primers for nested PCR and conditions were as before (67).

### Transmission electron microscopy.

Transmission electron microscopy was used as described previously (68, 69). In brief, samples were fixed overnight at 4°C using 2.5% glutaraldehyde in PBS. Afterward, samples were postfixed with 1% OsO_4_ at 4°C for 2 h, followed by serial ethanol dehydration and embedding in Epon 812 resin. Serial sections of uniform thicknesses (∼60 nm) were made using a Leica EM UC7 ultramicrotome (Germany). Ultrathin sections were then loaded onto 100-mesh Cu grids and double stained with 2% uranyl acetate and lead citrate before observations were made using a JEM 1400Plus transmission electron microscope (Japan) at 120 kV.

### Degradation of tsAPOBEC3 by Vif.

The pcDNA3.1-Vif-HA or the pcDNA3.1-Vif-SLQ-AAA-HA and pcDNA3.1-tsAPOBEC3-Flag plasmids were transfected at 5:1 into 293T cells. At 48 hpi, the cells were collected, and Vif and tsAPOBEC3 proteins were detected by Western blot analysis.

### Statistical analysis.

Data are presented as mean ± standard deviation (SD). Differences between groups were analyzed using Student's *t* test. Significant differences were scored as *, *P < *0.05; **, *P < *0.01; and ***, *P < *0.001.

### Data availability.

All sequences reported in this paper were deposited in GenBank as follows: tree shrew CD4, accession no. MN227147; tree shrew CCR5, accession no. MN227148; and tree shrew CXCR4, accession no. MN227149.
